# Oculomotor behavior tracks the effect of ideological priming on deception

**DOI:** 10.1038/s41598-020-66151-1

**Published:** 2020-06-12

**Authors:** Michael Schepisi, Giuseppina Porciello, Salvatore Maria Aglioti, Maria Serena Panasiti

**Affiliations:** 1 Department of Psychology, “Sapienza” University of Rome, Rome, Italy; 2 IRCCS, Santa Lucia Foundation, Rome, Italy; 3 Department of Psychology, “Sapienza” University of Rome and CNLS@Sapienza Istituto Italiano di Tecnologia, Rome, Italy

**Keywords:** Psychology, Human behaviour

## Abstract

The decision to lie to another person involves a conflict between one’s own and others’ interest. Political ideology may foster self-promoting or self-transcending values and thus may balance or fuel self vs. other related conflicts. Here, we explored in politically non-aligned participants whether oculomotor behavior may index the influence on moral decision-making of prime stimuli related to left and right-wing ideologies. We presented pictures of Italian politicians and ideological words in a paradigm where participants could lie to opponents with high vs. low socio-economic status to obtain a monetary reward. Results show that left-wing words decreased self-gain lies and increased other-gain ones. Oculomotor behavior revealed that gazing longer at politicians’ pictures led participants to look longer at opponent’s status-related information than at game’s outcome-related information before the decision. This, in turn, caused participants to lie less to low status opponents. Moreover, after lying, participants averted their gaze from high status opponents and maintained it towards low status ones. Our results offer novel evidence that ideological priming influences moral decision-making and suggest that oculomotor behavior may provide crucial insights on how this process takes place.

## Introduction

Deciding whether to deceive or not another person activates a motivational conflict between the temptation to lie in order to obtain a personal benefit and the desire to behave according to social norms^1^. Thus, this type of decision is not a static and univocal process, rather the result of an interplay between situational, dispositional and emotional factors^2–4^. On the one hand, the temptation to act dishonestly is enhanced by anonymity^5^, monetary priming^6^, unfavorable situations^2^, sense of entitlement^7,8^, activation of positive self-concepts^9,10^ and perception of game partners^11^. On the other hand, honest behavior increases when reputation is at risk^2,11,12^, or when the concept of honesty is made salient^1,13,14^.

Importantly, the decision to deceive depends on the centrality of moral values for each individual^15^. In this vein, political ideology can serve as a social regulator of morality^16,17^, by determining which values people prioritize. Specifically, right-wing people endorse values related to self-enhancement (like power and achievement), while left-wing people endorse values related to self-transcendence (like benevolence and universalism)^18,19^. While a few studies reported effects of political ideology on social decisions such as trust and cooperation^20–22^, to the best of our knowledge only a study (in the French context) investigated whether priming political vs. non-political dimensions differentially influenced cheating behavior^23^.

To understand whether and how political information can affect deceptive behavior, we combined an adapted version of a card game that investigates spontaneous deception (i.e., the Temptation to Lie Card Game, TLCG^2^) with a priming procedure based on images of political leaders and ideological words.

It is worth noting that contemporary political propaganda uses both ideological words and politicians’ images to influence voters’ behavior. In fact, because of the so-called “*personalization of politics*”, the image of a political leader seems to have become more prominent than parties and collective identities^24,25^ in influencing voters’ implicit social attitudes more than parties related cues^21,26–29^. When a word is categorized as right or left-wing, it can convey political messages with very little ambiguity as it clearly represents only one of the two polarized ideologies. This effect can be less strong with politicians’ faces categorized as right or left-wing. Indeed, given the increasing perception among people that politicians are corrupt and dishonest regardless of their political partisanship (see *Trendsetting* survey, SWG-Affaritaliani.it, 2010 and Istituto Demopolis, 2017), their faces can work as a prime for dishonesty irrespectively of whether they are left- or right-wing politicians. For this reason, we hypothesized that ideological words would affect deceptive behavior according to the associated ideology (see below for the specific hypothesis on different ideologies), while the images of both left- and right-wing politicians would increase dishonesty more than ideological words. We called this hypothesized phenomenon the “*caste effect*”.

Ideology-based moral differences have important implications on how social and economic inequality is addressed. In fact, according to the System Justification Theory^30^, right-wing ideology endorses the acceptance and legitimation of inequalities by supporting the existing order. Conversely, left-wing ideology tends to object social disparity by promoting values such as social change and resource redistribution^30^. In view of this, we added to the classical version of our game an information related to the socio-economic status of participants’ opponents. We expected that right-wing primes would induce an equal level of deception toward high and low-status opponents, while left-wing primes would enhance deception toward high-status ones.

Previous research has shown that oculomotor behavior provides valuable information about the process of decision-making at large^31^, and in particular in the domain of moral decisions. For example, people tend to allocate their attentive resources by avoiding undesirable information, such as those enhancing guilt, anxiety and cognitive dissonance^32–37^. Thus, in the present study we recorded participants’ fixations duration during specific moments of the decisional process. In fact, before deciding to lie or not, participants were simultaneously presented with two crucial pieces of information, one related to the outcome of the game (henceforth referred to as *personal* information, i.e. participants’ gain/loss) and the other to the status of the opponent (henceforth referred to as *social* information). Since left-wing ideology conveys concepts of equality and social support^30^, we expected left-wing primes to drive participants’ attention toward the social information. Conversely, when primed with a right-wing stimulus, we expected participants to direct their attention toward the personal information, as right-wing ideology seems to be associated with individualism and autonomy^30,38,39^. In turn, we hypothesized that participants who looked more at the opponent’s social status would make less self-gain lies and more other-gain lies to low-status opponents, and those who looked more at the outcome of the game would produce more self-gain lies regardless of their deception target.

Importantly, after each trial, we presented participants with a picture of their opponents and measured how long they would look at their eyes, which are likely the most important source of information during interaction and communication^40^.

After behaving in ways that lead to guilt, people react to the resultant arousal by avoiding eye contact with the target of their behavior^41^. Accordingly, we expected participants to look less at their opponent’s eyes after having lied, especially when facing low-status opponents. We expected this effect to be stronger when participants were primed with a left-wing stimulus because concepts evoked by this ideology, such as resource redistribution and social support, should induce participants to experience more cognitive dissonance after their immoral action^42^.

Finally, to explore whether inter-individual differences in personal dispositions could modulate these processes we measured two personality traits previously associated with deception, namely machiavellianism and social desirability^2,12,43^.

It is worth mentioning already at this point that we tested participants with no clearly defined political orientation. The choice was motivated by i) experimental evidence indicating that ideologically polarized people – particularly Conservatives – tend to resist change^44^, ii) the fact that our priming procedure may be too weak to induce a sufficient modulation in the behavior of polarized voters, and iii) the relevance of these electors in deciding the destiny of elections.

## Materials and Methods

### Participants

Based on the effect sizes obtained in previous research where the same experimental paradigm and similar manipulations were used (R^2^ = 0.43) (e.g.^11^), we performed a power analysis (power = 0.80; α = 0.05) for multiple regression (*pwr* R package) that estimated a sample of N = 39 to be adequate. We collected data from fifty Italian participants (26 females, age M = 23.04; SD = ± 3.13) to guard against possible participants’ exclusion due to the effectiveness of the cover story (see Procedure section) and possible data loss due to technical issues. Indeed, ten participants were excluded because they did not believe the cover story (see Supplementary materials for a detailed description) and one participant was discarded because of loss of eye tracker signal. Thus, the final sample consisted in thirty-nine participants (23 females, age M = 22.71; SD = ± 2.91) with normal or corrected to normal vision. All 50 participants (including those discarded) were chosen as having no clearly defined political orientation (i.e., those who described themselves as “*Neither left-wing nor right-wing”* on a 5-point self-placement scale ranging from “*left-wing*” to “*right-wing*”).

The experimental procedures were approved by the independent Ethics Committee of the Santa Lucia Foundation in Rome (Scientific Institute for Research Hospitalization and Health Care) and were in accordance with the 1964 Declaration of Helsinki. Subjects read and signed the informed consent and were paid a fixed amount of 15 euro for their participation. An additional, variable amount (between 2.50 and 10 euro) was given according to what they gained during the game (see Supplementary materials for payment procedure).

### Stimuli

Prime stimuli were seven pre-validated images of Italian politicians and words representing left and right-wing ideologies (for the detailed validation procedure see Supplementary materials). To strengthen the cover story we inserted as prime stimuli also some images of famous people not belonging to any political category and not ideologically-related words (see Supplementary materials). Sixteen images of unknown persons (8 males, 8 females) were also chosen to represent the fictitious opponents in the TLCG and assigned to high-status or low-status opponents group according to a counterbalanced order.

All stimuli were resized and presented in the adapted version of the TLCG^2^ using E-Prime 2.0 (Psychology Software Tool, PA).

### Main experimental task

#### The Temptation to Lie Card Game (TLCG)

The *Temptation to Lie Card Game (TLCG)* implies that two players interact in a card game. One player is the participant, while the other is the opponent. The opponent starts by picking one of two covered cards that can be either a winning or a losing card, without knowing which card s/he picked. The participant is then shown with the picked card and asked to communicate the outcome of the game to the opponent. In so doing, the participant can decide whether to lie or tell the truth, thus changing or not the original outcome of the game. This paradigm is a zero-sum game in which only one of the two players can obtain the unknown and fixed monetary reward associated to each winning trial.

Importantly, the TLCG presents two possible situations to the participant. In one case, when the opponent picks the winning card, the situation is *unfavorable* for the participant, and thus lying produces a *self-gain lie* that leads to a personal reward. Conversely, when the opponent picks the losing card, the participant faces a *favorable* situation, and lying becomes an *other-gain lie* that leaves the reward with the opponent.

**Figure 1:**
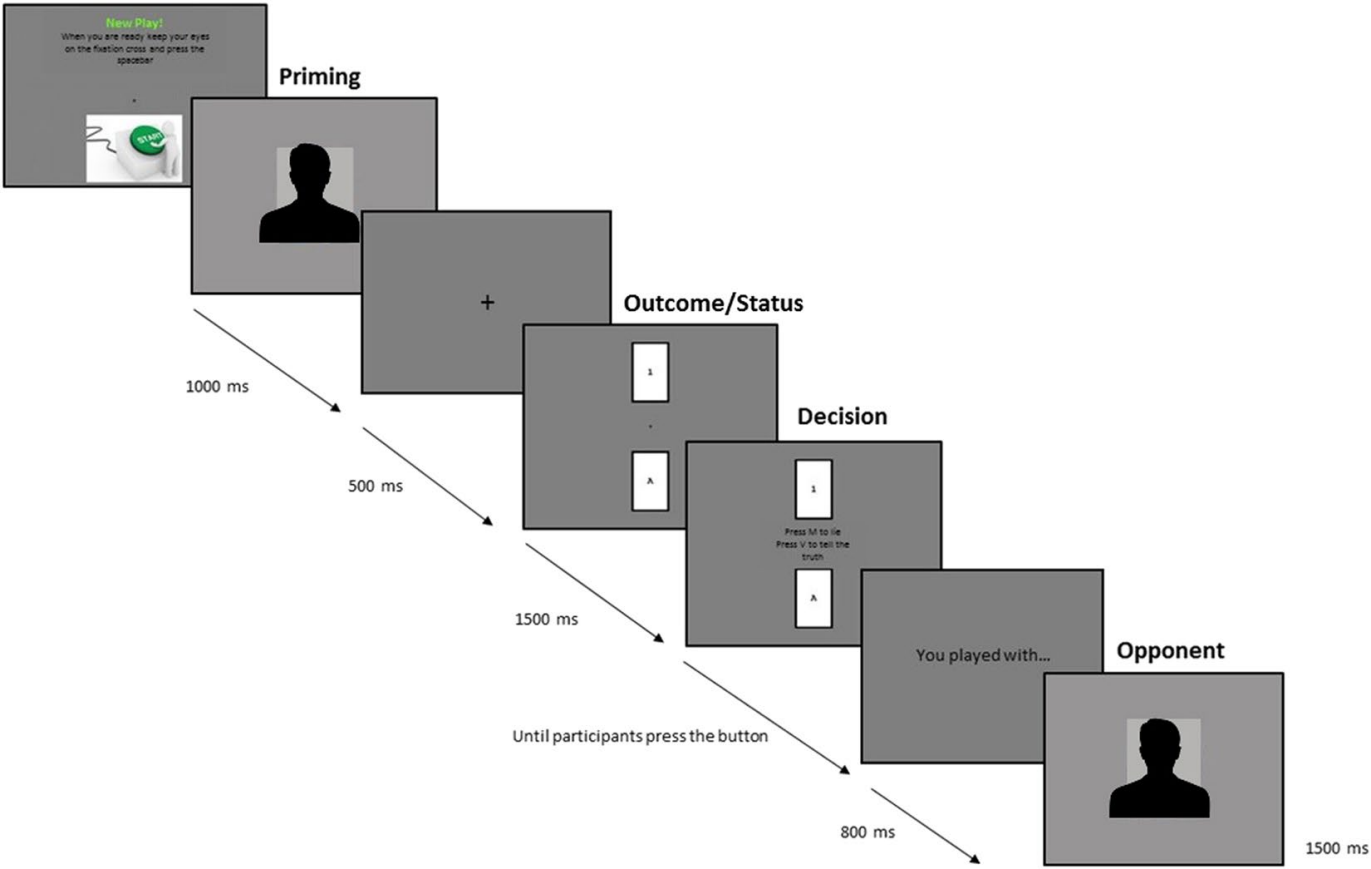
Exemplary timeline trial of the adapted version of the TLCG with a politician as prime stimulus. At the beginning of each trial, participants started to play by pressing the spacebar, which caused the prime stimulus to appear on the screen for 1000 ms. The stimulus could either be the image of a politician (366 × 492 pixels) or an ideological word (685 × 180 pixels). A fixation cross (500 ms) appeared before the card (190 × 300 pixels) referred to the outcome of the game and the card (190 × 300 pixels) representing the opponent's status were revealed. The two cards stayed on the screen for 1500 ms before the participants were able to decide whether to lie or tell the truth to the opponents. They did so by pressing either the “M” or “V” buttons on the keyboard, respectively. There was no time limit to make this choice. Then, 800 ms after deciding, the picture of the opponent appeared on the screen, where it stayed for 1500 ms. In the present figure the images of the politician and the opponent are presented anonymously. The figure was created by the authors.

For the purposes of our study, here we modified the original paradigm by i) adding one card symbolizing the opponent’s status and presented it simultaneously with the other card related to the outcome of the game, and ii) changed the symbols on the card with 1 or B vs. 9 or A that represented the equivalent of the original spade (loss) and heart (win) cards, respectively (see Fig. 1).

### Design

The study consisted of four blocks presented in an incomplete counterbalanced order (for the detailed procedure see Supplementary materials). Each block had 64 trials presented in a randomized order, for a total of 256 trials. Participants played 50% of trials against high-status opponents and 50% against low status-ones. Similarly, 50% of the trials presented favorable and the other 50% unfavorable situations. Political category (i.e., *left vs. right-wing*) of the prime stimulus was counterbalanced across blocks.

### Procedure

Participants began by sitting in front of an LCD monitor (1280 × 1024 resolution) placed 60 cm from their eyes. Then they received information about the experiment, signed the consent form and were instructed about the experiment. We told them that the experiment would consist in playing a card game with some employees of a big company whose responses had been previously recorded (i.e., the cover story; see Supplementary materials). We then described the meaning of the symbols on the cards and presented the participants with two brief descriptions of their opponents: high status opponents were managers with a permanent position at the company, while low status opponents were office workers with a fixed-term contract.

Participants performed a brief practice session consisting of six trials. Then the experimenter turned off the light and began the standard 9-points eye tracker’s calibration procedure, which was followed by a more accurate one where participants were asked to explore colored geometric shapes placed in the position of the Areas of Interest (AOI) drawn for the TLCG. Both procedures were performed before starting each of the four experimental blocks. We recorded participants’ oculomotor behavior monocularly at a sampling rate of 250 Hz by means of an head-free infrared video-based eye-tracking system (SMI RED 500, Sensomotoric Instruments, GmbH, Germany). To prevent any issue with this specific recording system and procedure, we asked participants to stay as still as possible and reminded them this instruction every time we noticed loss of eye-tracking signal due to their excessive movements.

Before starting the experimental task, the images of the sixteen opponents were presented to participants in a random order. This was done to obtain a baseline measure of participants’ oculomotor behavior that could then be compared to the one measured later during the TLCG.

Participants then performed the adapted version of the TLCG (a detailed description of a typical experimental trial is provided in Fig. 1). At the end of the TLCG, participants were asked to respond to the following questions to determine whether they had believed the cover story: i) “*did you feel involved in the game?”* (“yes” or “no”) and ii) “*how involved did you feel*?” (5-point scale with 1 being “not at all” and 5 “very much”).

Finally, participants performed a recognition and categorization task of the prime stimuli (see Supplementary materials), and completed personality questionnaires measuring Social Desirability^45^ and Machiavellianism^46^.

### Data handling

For each trial of the TLCG we analyzed: i) participants’ behavioral responses related to the tendency to lie (i.e., lie vs. truth) and ii) participants’ oculomotor behavior (i.e., duration of the fixations spent on each selected AOI). The duration of the fixations spent in each of the targeted AOI (see Supplementary materials) was measured in three specific moments during the trial, namely when participants were presented with: i) the *prime stimulus*, ii) the two cards representing the *outcome of the game* and the *opponent’s status*, iii) the picture of the *opponent*. Fixations duration was then extracted using the software BeGaze 3.6 (SMI, Sensomotoric Instruments, GmbH, Germany).

### Data analysis

Data analysis was performed using the statistical software R^47^. We used the package *lme4* Version 1.1–5^48^ to run multilevel mixed linear regression (LMM or “linear mixed-effects models”) and multilevel log-linear regression analyses (GLMM, “generalized mixed-effects models”)^49,50^ for continuous and dichotomous dependent variables respectively.

The random effects structure of each statistical model was built by means of a Principle Components Analysis (PCA; R package *RePsychLing*, function *rePCA*), an analysis that tests the over-parameterization of the maximum random structure, something that can affect the interpretability and reliability of the parameters’ estimates^51^. PCA reveals the amount of variance captured by each random effect. Thus, by-subject random effects that explained zero amount of variance were removed. FDR-corrected post-hoc comparisons were performed with the R package *lsmeans*. Type III Wald *Anova* function from the R package *car* was used to determine the statistical significance of the fixed effects.

## Results

### Factors influencing the tendency to lie

We ran a multilevel mixed log-linear regression analysis on the model with the *Tendency to lie* as dependent dichotomous variable (i.e., lie/truth) and *Priming type* (ideological words vs. politician pictures), *Political orientation of the priming* (left vs. right-wing), *Outcome of the game* (favorable vs. unfavorable), *Opponent’s Status* (low vs. high) and their respective interactions as fixed effects (for the list of significant and non-significant effects and descriptive statistics see Tables 1, 2, 3, 4, 5; for the syntax see model 1 in Supplementary materials).

**Table 1:** Effects related to how experimental factors influence deceptive behavior.

	Χ^2^	p
Intercept	9.4164	0.002***
Political Orientation of the Priming	3.2016	0.073
Opponents’ Status	13.7616	0.000***
Priming Type	0.1971	0.657
Outcome of the game	0.6039	0.437
Political Orientation of the Priming*Opponents’ Status	0.1276	0.720
Political Orientation of the Priming*Priming Type	0.9057	0.341
Opponents’ Status*Priming Type	0.6705	0.412
Political Orientation of the Priming*Outcome of the game	5.0632	0.024*
Opponents’ Status*Outcome of the game	23.1529	0.000***
Priming Type*Outcome of the game	0.1858	0.666
Political Orientation of the Priming*Opponents’ Status*Priming Type	0.0123	0.911
Political Orientation of the Priming*Opponents’ Status*Outcome of the game	0.0151	0.902
Political Orientation of the Priming*Priming Type*Outcome of the game	5.1844	0.022*
Opponents’ Status*Priming Type*Outcome of the game	2.9264	0.087
Political Orientation of the Priming* Opponents’ Status* Outcome of the game* Priming Type	0.6542	0.418

**Table 2:** Effects related to how experimental factors and priming fixations duration influence oculomotor behavior toward Social vs. Personal information.

	Χ^2^	p
Intercept	0.7042	0.401
Priming Fixations Duration	3.4644	0.062
Priming Type	2.9273	0.087
Political Orientation of the Priming	1.6827	0.194
Priming Fixations Duration* Priming Type	2.8963	0.088
Priming Type* Political Orientation of the Priming	0.5786	0.446
Priming Type*Political Orientation of the Priming	0.0224	0.880
Priming Fixations Duration *Priming Type*Political Orientation of the Priming	4.6101	0.031*

**Table 3:** Effects related to how experimental factors and the tendency to look at personal information first influence deceptive behavior.

	Χ^2^	p
Intercept	9.9927	0.001***
Opponents’ Status	61.8734	0.000***
Outcome of the game	0.7171	0.397
Personal First	0.5045	0.477
Opponents’ Status*Outcome of the game	419.3762	0.000***
Opponents’ Status*Personal First	0.2509	0.616
Outcome of the game*Personal First	7.5152	0.006***
Outcome of the game*Personal First*Opponents’ Status	6.2441	0.012*

**Table 4:** Effects of the experimental factors and deceptive behavior on the oculomotor behavior toward the opponents.

	Χ^2^	P
Intercept	0.9018	0.342
Lie/Truth	1.1361	0.286
Outcome of the game	0.2162	0.641
Opponents’ Status	0.0445	0.833
Political Orientation of the Priming	0.0432	0.835
Priming Type	0.4868	0.485
Lie/Truth*Outcome of the game	1.0669	0.301
Lie/Truth*Opponents’ Status	4.7130	0.029*
Outcome of the game*Opponents’ Status	0.6625	0.415
Lie/Truth*Political Orientation of the Priming	0.0427	0.836
Outcome of the game* Political Orientation of the Priming	0.0012	0.972
Opponents’ Status* Political Orientation of the Priming	1.4356	0.230
Lie/Truth*Priming Type	0.4094	0.522
Outcome of the game* Priming Type	0.7925	0.373
Opponents’ Status* Priming Type	3.8353	0.050
Political Orientation of the Priming*Priming Type	1.1941	0.274
Lie/Truth*Outcome of the game*Opponents’ Status	3.4636	0.062
Lie/Truth*Outcome of the game*Political Orientation of the Priming	0.0745	0.784
Lie/Truth*Opponents’ Status*Political Orientation of the Priming	1.2582	0.261
Outcome of the game*Opponents’ Status*Political Orientation of the Priming	0.4557	0.499
Lie/Truth*Outcome of the game*Priming Type	0.4523	0.501
Lie/Truth*Opponents’ Status*Priming Type	4.8227	0.028*
Outcome of the game*Opponents’ Status*Priming Type	2.6729	0.102
Lie/Truth*Political Orientation of the Priming*Priming Type	0.5470	0.459
Outcome of the game*Political Orientation of the Priming*Priming Type	0.2898	0.590
Opponents’ Status*Political Orientation of the Priming*Priming Type	0.2370	0.626
Lie/Truth*Outcome of the game*Opponents’ Status*Political Orientation of the Priming	1.3539	0.244
Lie/Truth*Outcome of the game*Opponents’ Status*Priming Type	4.0973	0.042*
Lie/Truth*Outcome of the game*Priming Type*Political Orientation of the Priming	0.1007	0.750
Lie/Truth*Priming Type*Opponents’ Status*Political Orientation of the Priming	1.6265	0.202
Outcome of the game*Opponents’ Status*Political Orientation of the Priming*Priming Type	0.0354	0.850
Lie/Truth*Outcome of the game*Opponents’ Status*Political Orientation of the Priming*Priming Type	0.7670	0.381

**Table 5:** Percentages of lie vs. truth decisions depending on the different experimental conditions.

Lies vs Truths	
Total Lies	31%
Total Truths	69%
Self-gain Lies	20.66%
Other-gain Lies	10.37%
Self-gain Lies toward High Status Opponents	12.83%
Self-gain Lies toward Low Status Opponents	7.83%
Other-gain Lies toward High Status Opponents	3.57%
Other-gain Lies toward Low Status Opponents	6.80%
Self-gain Lies with a Right-wing Word prime	5.35%
Self-gain Lies with a Left-wing Word prime	4.92%
Other-gain Lies with a Right-wing Word prime	2.90%
Other-gain Lies with a Left-wing Word prime	3.08%
Self-gain Lies with a Right-wing Politician prime	5.13%
Self-gain Lies with a Left-wing Politician prime	5.23%
Other-gain Lies with a Right-wing Politician prime	2.31%
Other-gain Lies with a Left-wing Politician prime	2.07%

**Figure 2:**
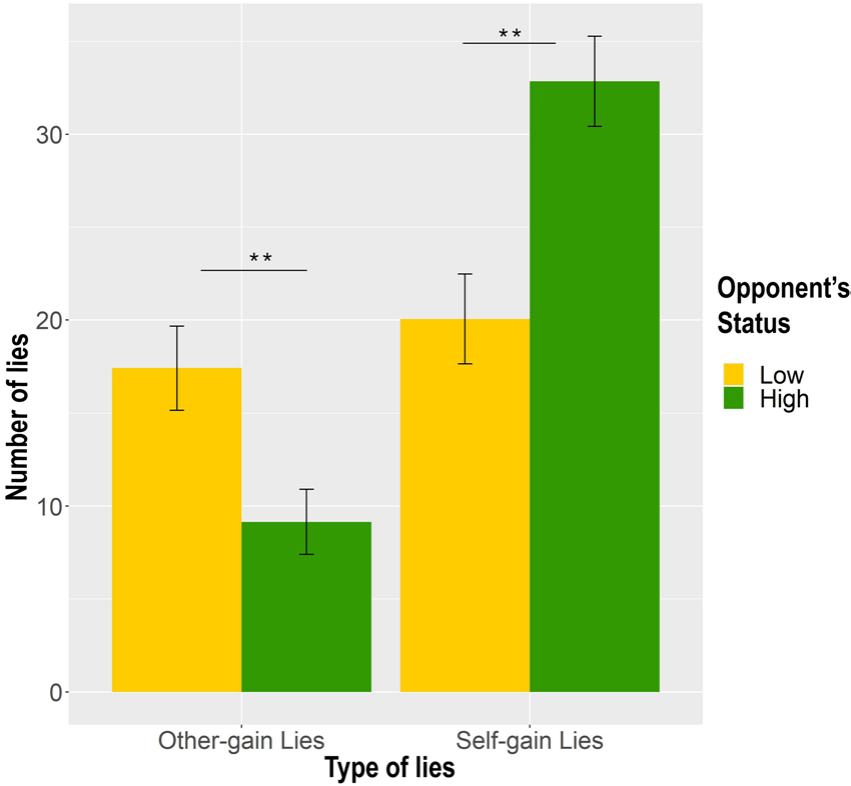
Factors influencing deceptive behavior. The figure shows the modulation of the *deceptive behavior, i.e., number of lies* (on the Y-axis) depending on the *Outcome of the game* and *Opponent’s Status*. On X-axis lies are divided in: other-gain when the outcome of the game is favorable for the participant, self-gain when it is unfavorable. Participants produced more other-gain lies when facing a low status opponent and more self-gain lies when facing a high-status opponent. **p < 0.01; *p < *0.05*. The figure was made in R using ggplot2 package^76^.

**Figure 3:**
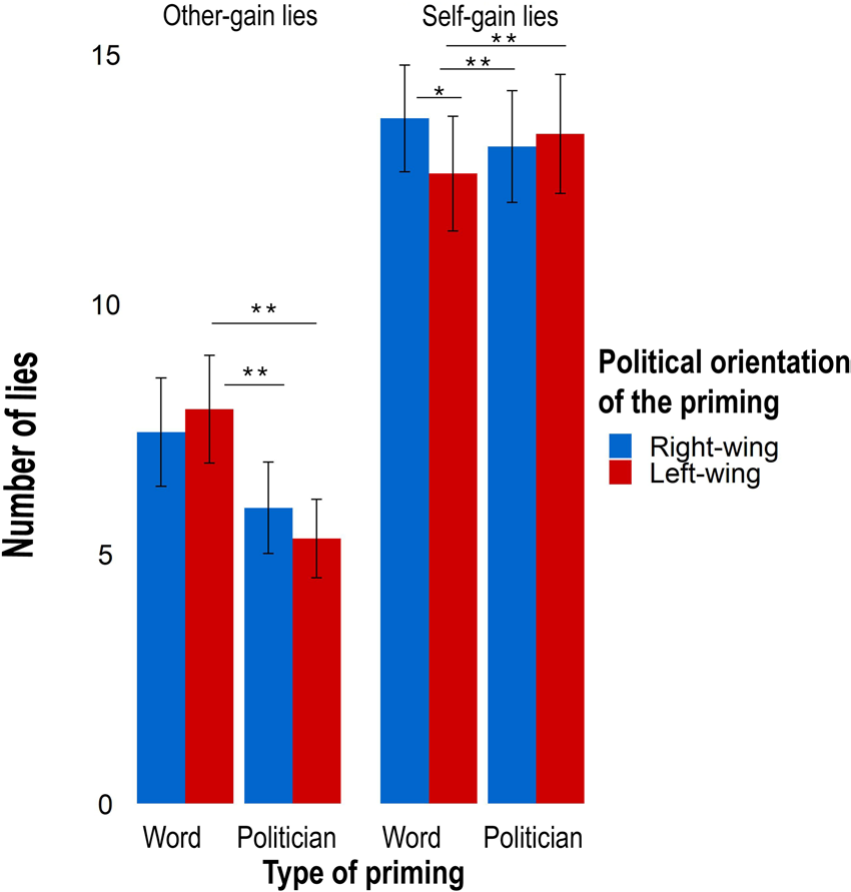
Factors influencing deceptive behavior. The figure shows the effect on deceptive behavior of the *Priming type* (politician pictures vs. ideological words) and *Political Orientation of the priming* (left-wing vs. right-wing) depending on the *Outcome of the game*. The left grid shows the number of lies produced when the outcome of the game was favorable for the participant (other-gain lies), the right grid when the outcome was unfavorable (self-gain lies). **p < 0.01; *p < *0.05*. The figure was made in R using ggplot2 package^76^.

The model (R^2^_marginal_ = 0.178, R^2^_conditional_ = 0.639) showed a significant *Outcome of the game* X *Opponent’s Status* interaction (estimate = 2.778, SE = 0.577, χ^2^ = 23.152, p < 0.001, lower CI = 1.646, upper CI = 3.910) that revealed the role of the status of the opponent in the decision to lie for personal vs. altruistic purposes. Post-hoc comparisons showed that participants tended to favour their opponents when these had a low socio-economic status by producing more other-gain lies (estimate = 1.440, SE = 0.300, z-ratio = 4.802, p < 0.001), while they tended to favour themselves when opponents had a high socio-economic status by producing more self-gain lies (estimate = −1.553, SE = 0.295, z-ratio = −5.264, p < 0.001) (see Fig. 2). Importantly, the role of political priming in this process was qualified by the significant *Priming type* X *Political Orientation* o*f the priming* X *Outcome of the game* interaction (estimate = 0.723, SE = 0.317, χ^2^ = 5.184, p = 0.02, lower CI = 0.100, upper CI = 1.347), which however did not take into account the socio-economic status of the opponents (estimate = 0.039, SE = 0.323, χ^2^ = 0.015, p = 0.90, lower CI = −0.594, upper CI = 0.674). Post-hoc comparisons revealed that participants produced more other-gain lies when primed with left-wing words than with pictures of left-wing politicians (estimate = 0.446, SE = 0.162, z-ratio = 2.755, p = 0.009, lower CI = 0.128, upper CI = 0.763) and of right-wing politicians (estimate = 0.479, SE = 0.180, z-ratio = 2.656, p = 0.01, lower CI = 0.126, upper CI = 0.831). They also produced more self-gain lies when primed with right-wing words with respect to left-wing words (estimate = 0.238, SE = 0.113, z-ratio = 2.108, p = 0.04, lower CI = 0.016, upper CI = 0.459), and when primed with pictures of left (estimate = −0.474, SE = 0.144, z-ratio = −3.277, p = 0.002, lower CI = −0.756, upper CI = −0.191) and of right-wing (estimate = −0.437, SE = 0.133, z-ratio = −3.280, p = 0.002, lower CI = −0.697, upper CI = −0.176) politicians with respect to left-wing words. Confirming our hypothesized “*caste effect*” we found no differences in how left and right-wing politicians primes affected participants’ tendency to lie (estimate = −0.037, SE = 0.155, z-ratio = −0.0239, p = 0.82, lower CI = −0.340, upper CI = 0.266) (see Fig. 3).

### Oculomotor behavior and its effects on deceptive responses

**Figure 4:**
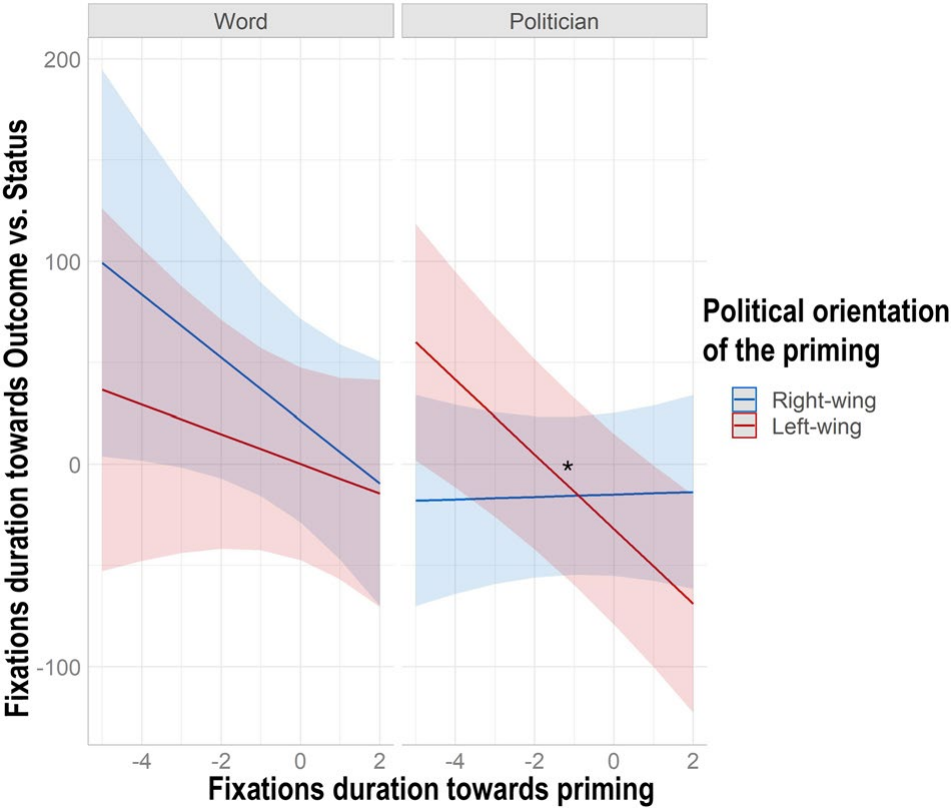
Oculomotor behavior related to the preference for social vs. personal information. The figure shows the effect of the fixations duration of the political priming on attention allocation towards the outcome of the game vs. the status of the opponent. The X-axis shows the amount of time spent looking at the priming (Priming Fixations Duration) centered to the mean. The Y-axis shows the ratio of the fixations duration towards the card depicting the Outcome to that depicting the Status of the opponent (*Personal First*). Results indicated that the more participants looked at left-wing politicians, the more their gaze shifted towards the social information (i.e., Status of the opponent). The figure was made in R using ggplot2 package^76^.

To test how political priming affected participants’ oculomotor behavior and the subsequent decision to lie we ran a multilevel mixed linear regression on the ratio relative to the time spent fixating the “*Outcome AOI*” to the “*Status AOI*”. Higher values for this ratio indicated that participants spent more time looking at the personal compared to the social information (*Personal first*). Since, in principle, the scan path of images of politicians vs. ideological words requires different exploration patterns, we transformed the fixations duration of the two prime stimuli in zeta scores and created a new regressor called *Priming Fixations Duration*. Thus, *Priming Fixations Duration*, *Priming type, Political Orientation* o*f the priming* and their respective interactions were the fixed effects of our model (see Tables 2 and 6; for the syntax see model 2 in Supplementary materials). Confirming our predictions, the model (R^2^_marginal_ = 0.004, R^2^_conditional_ = 0.218) showed a significant *Priming Fixations Duration* X *Priming type* X *Political Orientation of the priming* interaction (estimate = −27.298, SE = 12.714, ^2^ = 4.610, p = 0.03, lower CI = −52.217, upper CI = −2.379). To qualify the significant interaction of this regression model we tested its simple effects using the *simple slopes* function included in the R package *reghelper*. The analysis indicated that the longer participants looked at a left-wing prime, the more they directed their attention toward the social information (i.e., opponent’s status). In contrast with our hypotheses, this occurred only for left-wing politicians (b = 18.443, SE = 4.621, t-value = 3.990, upper CI = −9.385, lower CI = −27.501), while left-wing words did not show any effect (b = −7.305, SE = 7.716, t-value = −0.946, upper CI = 9.515, lower CI = −22.430). Similarly, neither right-wing politicians (b = 0.613, SE = 4.542, t-value = 0.134, upper CI = 9.515, lower CI = −8.289) nor right-wing words (b = −15.547, SE = 8.353, t-value = −1.861, upper CI = 0.824, lower CI = −31.919) had any effect (see Fig. 4).

**Table 6:** Fixation time (in milliseconds) spent on each AOI depending on deceptive behavior and experimental conditions.

Outcome AOI vs. Status AOI	Milliseconds
Outcome AOI before Self-gain Lies	306.81 ms
Status AOI before Self-gain Lies	255.90 ms
Outcome AOI before Other-gain Lies	295.51 ms
Status AOI before Other-gain Lies	254.92 ms
Outcome AOI before Self-gain Lies toward High Status Opponents	301.65 ms
Status AOI before Self-gain Lies toward High Status Opponents	258.21 ms
Outcome AOI before Self-gain Lies toward Low Status Opponents	311.97 ms
Status AOI before Self-gain Lies toward Low Status Opponents	253.58 ms
Outcome AOI before Other-gain Lies toward High Status Opponents	291.85 ms
Status AOI before Other-gain Lies toward High Status Opponents	271.27 ms
Outcome AOI before Other-gain Lies toward Low Status Opponents	299.15 ms
Status AOI before Self-gain Lies toward Low Status Opponents	238.59 ms

**Figure 5:**
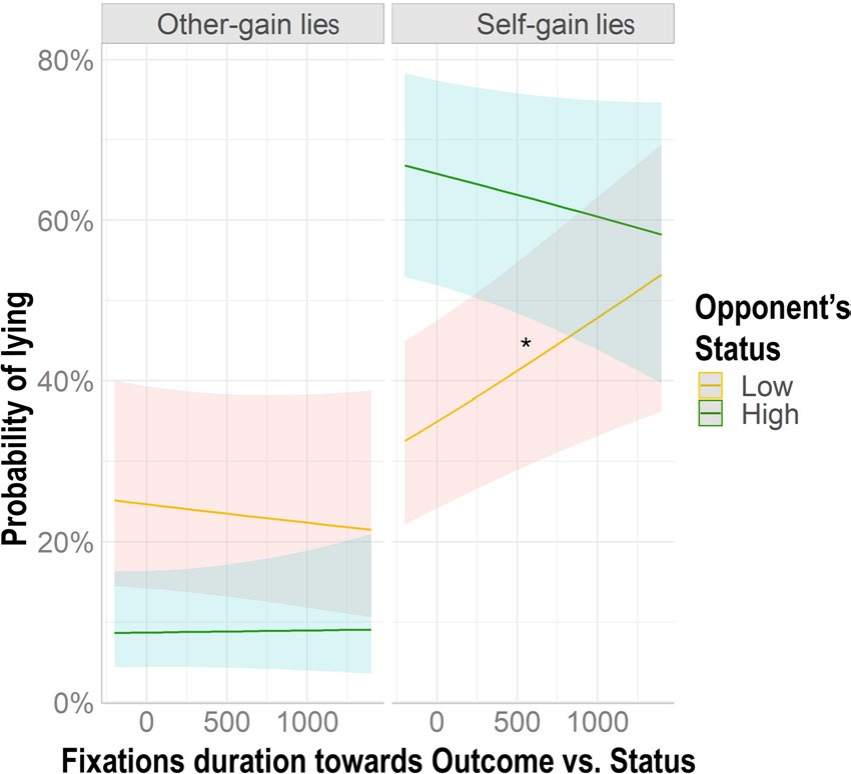
The influence of oculomotor behavior related to the preference for social vs. personal information on deceptive behavior. The figure shows the effect of attention allocation towards the social information following a left-wing politician on deceptive behavior. The X-axis shows fixations toward social vs. personal information (*Personal First*) centered to the mean. Moving towards the right side of the X-axis indicates fixation toward the Status of the opponent, moving on the left side indicates fixations toward the Outcome of the game. The more participants looked at the social information, the less they lied to low status opponents. The figure was made in R using ggplot2 package^76^.

Following this result, we tested: (i) whether paying more attention to social information would lead participants to make less self-gain lies and more other-gain lies toward low (vs. high) status opponents, and ii) whether paying attention to the personal information would lead them to produce more self-gain lies regardless of the status of the opponents. We ran a multilevel mixed log-linear regression with the *Tendency to Lie* (*Lie/Truth*) as our dependent variable and *Opponent’s Status*, *Outcome of the game*, the ratio between the fixations towards the outcome and the status (*Personal first*), and their respective interactions as our fixed effects (see Tables 1 and 5; for the syntax see model 3 in Supplementary materials). As predicted, the model (R^2^_marginal_ = 0.197, R^2^_conditional_ = 0.579) revealed a significant *Personal first* X *Opponent’s Status* X *Outcome of the game* interaction (estimate = −0.0009, SE = 0.0003, χ^2^ = 6.244, p = 0.01, lower CI = −0.0016, upper CI = −0.0001). We tested the simple slope of *Personal first* for the interaction of *Opponent’s Status* and *Outcome of the game* on the *Tendency to Lie*. Partially in line with our hypothesis, analysis revealed that the more participants looked at the social information (i.e., the card depicting the opponent’s status), the less they tended to produce self-gain lies directed to low-status opponents (b = 0.0005, SE = 0.0001, t-value = 3.195, upper CI = 0.0008, lower CI = 0.0002), but no effect on other-gain lies was found (b = −0.0001, SE = 0.0001, t-value = −0.710, upper CI = 0.0002, lower CI = −0.0004) (See Fig. 5). Moreover, and in contrast with our prediction, no effects were found for high-status opponents neither for self (b = −0.0002, SE = 0.0001, t-value = −1.335, upper CI = 0.0001, lower CI = −0.0005) nor for other-gain lies (b = 0.0002, SE = 0.0002, t-value = 0.108, upper CI = 0.0004, lower CI = −0.0004).

**Figure 6:**
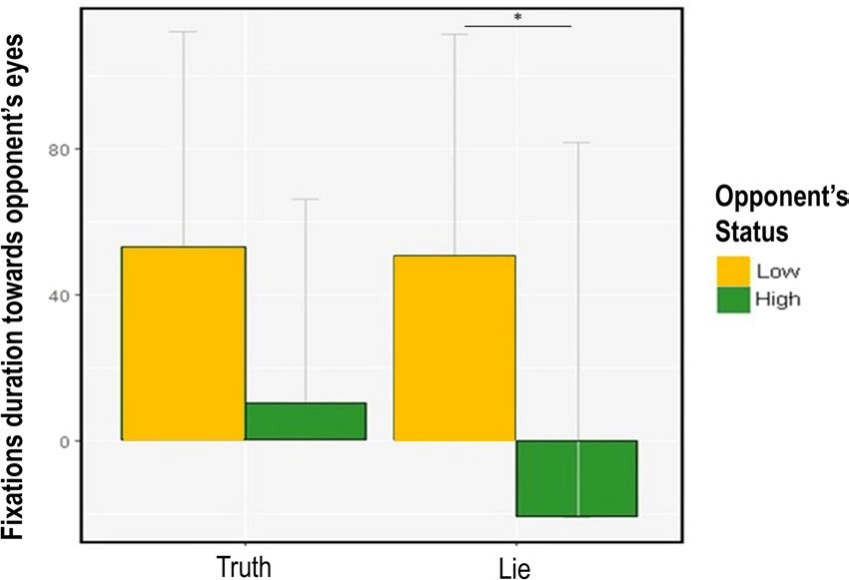
Oculomotor behavior towards opponents’ eyes. Figure 6 shows the effect of deceptive behavior on participants’ gaze toward their opponents’ eyes. The dependent variable was obtained by subtracting the fixation durations made by the participants toward the AOI containing the eyes of the opponents before the TLCG from those during the game. *p < 0.0*5*. The figure was made in R using ggplot2 package^76^.

For the last step of our analysis, we tested whether participants’ gaze toward their opponents was affected by their previous decision to lie and by the other factors tested in our experiment (i.e., Opponents’ status and Outcome of the Game). We obtained the dependent variable by subtracting participants’ fixations duration toward the AOI containing the eyes of the opponents taken before the beginning of the TLCG from that taken in the same AOI during the game. With *Opponent's Status*, *Outcome of the game*, *Lie/Truth, Priming type* and *Political Orientation of the priming* as our fixed effects, we ran a multilevel mixed linear regression on the baseline-corrected fixations toward the AOI that included the eyes of the opponent (*“Fixations towards opponents’ eyes”)* (see Table 4; for the syntax see model 4 in Supplementary materials). The model (R^2^_marginal_ = 0.006, R^2^_conditional_ = 0.460) showed a significant *Opponent’s Status X Lie/Truth* interaction (estimate = −158.866, SE = 73.178, χ^2^ = 4.713, p = 0.02, lower CI = −302.292, upper CI = −15.439). Post-hoc comparisons revealed that, after lying, participants tended to avert their gaze from high-status opponents and to maintain their gaze on low-status opponents (estimate = 71.832, SE = 28.110, z-ratio = 2.555, p = 0.04, lower CI = 16.736, upper CI = 126.927). Conversely, no effect was found when participants told the truth (estimate = 42.707, SE = 26.573, z-ratio = 1.607, p = 0.16, lower CI = −9.376, upper CI = 94.790) (see Fig. 6). Because the *Opponent’s Status X Lie/Truth X Outcome of the game* was not significant (estimate = 187.311, SE = 100.646, χ^2^ = 3.463, p = 0.06, lower CI = −9.952, upper CI = 384.574) we cannot say whether participants’ gaze aversion was specific for the type of lie they made (i.e., self vs. other-gain lie). The interaction with *Political Orientation of the priming* was not significant (estimate = −155.311, SE = 133.476, χ^2^ = 1.353, p = 0.24, lower CI = −416.919, upper CI = 106.297). We found a significant *Opponent's Status X Lie/Truth X Priming type* interaction (estimate = −286.972, SE = 141.771, χ^2^ = 4.097, p = 0.04, lower CI = −564.838, upper CI = −9.105), but it was not qualified by any significant post-hoc comparison (z-ratios < 2.976, ps > 0.17).

### Personality traits

**Figure 7:**
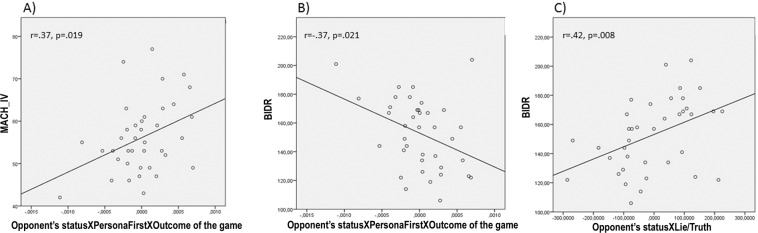
Correlations between personality traits and BLUPs of significant interactions. (**A**) This panel shows the association between the measure of Machiavellianism (MACH IV) and the *Opponent’s Status X Personal First X Outcome* interaction BLUPs on deceptive behavior. Higher scores in manipulative traits are associated with a weaker tendency to lie less to low status opponents after having looked more at the social than the personal information. (**B**) This panel shows the association between the BIDR scale and the *Opponent’s Status X Personal First X Outcome* interaction BLUPs on deceptive behavior. Higher scores in social desirability and impression management traits are associated with a stronger tendency to lie to low status opponents after having looked more at the social than the personal information. (**C**) This panel shows the association between the measure of social desirability (i.e., the Balanced Inventory of Desirable Responding, BIDR) and the BLUPs of the *Opponent’s Status X Lie/Truth* interaction on participants’ gaze towards the picture of the opponent. Higher scores in social desirability and impression management traits are associated with a stronger tendency to fixate low-status opponents after lying. The figure was made in SPSS^77^.

As exploratory analysis we correlated participants’ personality traits with their deceptive and oculomotor behavior during the TLCG. For each participant we extracted the individual-specific slope for each significant interaction (i.e., the so-called ‘BLUPs’, best linear unbiased predictors^52^) by using the *coef* function in R, which gives the fixed effect of that interaction plus the by-subject random effect. BLUPs for each interaction were correlated with participants’ scores on the Machiavellianism scale (MACH IV) and on the Balanced Inventory of Desirable Responding (BIDR). The correlations between the two personality measures and the *Opponent’s Status X Personal First X Outcome of the game* interaction BLUPs turned out to be significant. This interaction referred to how participants’ tendency to decrease their self-gain lies aimed at low-status opponents was affected by looking more at the social information than the personal one. Specifically, while a positive score refers to the tendency to look more at the Outcome of the game, a negative score refers to the tendency to look more at the Opponents’ status. Results revealed that the more participants were manipulative (i.e., higher scores in MACH IV scale), the less the preferential looking for the social information (positive scores) induced differences in the tendency to lie to low vs. high-status opponents (r(38) = 0.37, p = 0.019, two-tailed) (see Fig. 7, panel A). Conversely, the more participants gave importance to conveying a positive image of themselves (i.e., higher scores in BIDR), the more the preferential looking for the social information (negative scores) decreased deception toward low-status opponents (r(38) = −0.37, p = 0.021, two-tailed) (see Fig. 7, panel B).

Finally, we tested whether these personality traits were also associated with the tendency shown by participants to avert their gaze from the eyes of high-status opponents vs. maintain eye-contact with low-status ones after lying (the *Opponent’s Status* X *Lie/Truth* interaction on fixations duration). We observed no association with the Machiavellianism scale (r(38) = −0.07, p = 0.64, two-tailed). Conversely, the more participants were concerned about conveying a positive self-image, the less they tended to maintain eye-contact with low-status opponents (and the less they tended to avert their gaze from high-status ones) (r(38) = 0.42, p = 0.008, two-tailed) (See Fig. 7, panel C).

## Discussion

Using a modified version of an ecological paradigm for investigating situational and dispositional determinants of spontaneous lies (i.e., the TLCG^2^) we explored how different types of stimuli referring to different political ideologies affect the tendency to deceive a game partner perceived as low- or high-status. We used a priming procedure in which we exposed politically non-aligned people to images of politicians and words representing left and right-wing ideologies before deciding whether to lie or not to opponents with high vs. low socio-economic status. Moreover, to have a comprehensive understanding of how political priming affects information search and processing during moral decision-making, we recorded participants’ oculomotor behavior.

Expanding previous findings about how socio-economic status of interaction partners modulates altruism by inducing direct moral actions (i.e., donating money to low-status people; see^53,54^), we show that socio-economic status of interaction partners can trigger ostensible immoral actions that are altruistic indeed (i.e., other-gain lies made to change the unfavorable outcome for low-status people).

Importantly, we also demonstrate how political priming may contribute in directing and changing people’s deceptive behavior. In contrast with our hypothesis, results show that the effects of the priming were not dependent on the socio-economic status of the opponents. In fact, participants were in general more willing to produce other-gain lies when primed with left-wing words compared to pictures of left and right-wing politicians. Importantly, left-wing words also decreased the tendency to produce self-gain lies compared to the other prime stimuli. In situations in which the conflict between the self and other’s interest is particularly salient, social norms often play a major role by directing people’s behavior toward pro-sociality. A recent integrative model proposes that norm-based decisions arise from model-based computations that are integrated from the surrounding context and that change the value assigned to a certain action^55^. In particular, when deciding whether to deceive or not another person, people would experience a conflict between a model-free action value system (i.e., deception can lead to *obtain rewards*), which relies on meso-striatal structures and phasic dopamine signaling, and model-based representation system (i.e., information we learnt from the past: *people who deceive are bad*), which relies on prefrontal structures and tonic dopamine signaling^55^. In such vein, we suggest that, by referring to social norms that promote appropriate social interactions such as fairness, reciprocity and cooperation^56^, left-wing words might facilitate and reinforce the non-conscious activation of model-based representations of decision making, resulting in pro-social behavior.

Crucially, the absence of differences in the way left and right-wing politicians affected participants’ deceptive behavior confirms our hypothesized “*caste effect*”, namely the expression of a general mistrust towards the political system. This result in Italian voters follows what was recently found in a French study employing only pictures of politicians as prime stimuli^23^. The authors found that people tend to lie more when primed with stimuli representing politicians than when primed with non-politician stimuli (i.e., clergymen). In fact, while clergymen triggered a sort of moral reminder – something already seen to promote honesty^1^ – politicians may have triggered concepts of corruption that led participants to lie for personal purposes. This was confirmed by the fact that participants evaluated politicians as less honest and trustworthy than clergymen^23^.

By measuring participants’ oculomotor behavior we better characterized how ideological priming affects their decision to deceive. In contrast with our expectations, neither left- nor right-wing words exerted any effect on participants’ attention allocation toward the specific information provided. Importantly, we found that the more participants looked at left-wing politicians, the more they looked at the social information. In light of previous studies showing that right-wing politicians implicitly affect social behavior of voters, such as gaze following^27,28^ or trust^21^, we did not expect this strong modulation triggered by pictures of left-wing leaders compared to the other primes. This unexpected result could be explained by considering the political context during data collection. The present study was conducted while a coalition guided by a left-wing party was in power. This may have led our participants to perceive left-wing politicians as having a higher status and, thus, interfering on participants’ gaze. Indeed, past research showed that power affects basic and more complex behaviors^57^; for example, both human and non-human primates are strongly distracted by the gaze of high-status members^58,59^. Interestingly, the malleability of power and status is demonstrated in a recent study where more positive evaluation were given to unacquainted individuals who were assigned to a high-status category following a simple status-inducing strategy^60^.

Moreover, power affects people’s attentional focus by inducing them to selectively process environmental information in line with the demands of the tasks, especially when this information serves to achieve personal goals^61,62^. In this vein, we speculate that focusing on the social information could have been the best strategy for our participants to achieve their goals. Therefore, power-related stimuli -such as left-wing politicians- may have led participants to focus on that information.

Importantly, the attentional shift toward the social information led participants to lie less to low-status opponents, indicating that the greater was the focus on the social information, the less they lie to disadvantaged people.

Finally, we showed that participants engaged in two different but complementary actions after deceiving to their opponents: on the one hand, they tended to avert their gaze from the eyes of high-status opponents; on the other, they maintained eye contact with low-status ones. In a morally neutral situation, when no deception is involved, the attractive power of high-status individuals and leaders’ gaze is stronger compared to that of low-status individuals and followers^63–65^. However, in our study participants engaged in the immoral behavior of lying to their interaction partners, a condition that might have affected the usual observed behavioral pattern. To deal with this seeming discrepancy we propose two non-mutually exclusive explanations. The first is that gaze aversion toward high-status opponents could reflect participants’ guilt toward them^41,66^. The second is that lying to high status individuals might represent a social threat to which people react with submissive gaze aversion^67^. In fact, participants were aware that their opponents were informed about the results of the game and, thus, of the later consequences of their actions. Accordingly, Gobel and colleagues observed that participants tend to look less at high-status individuals when they believed that those targets could be later looking at them^68^.

As regard to personality, we found that high manipulative participants (i.e., high in Machiavellianism) tended to reduce less their deceptive behavior toward low-status opponents after looking at the social information. In keeping with studies where Machiavellians tend to lie more often^69^, more strategically^70^, in multiple contexts^71^ and without caring about possible reputational risks^2^, the above result may be explained by Machiavellians’ being prone to maximize their self-interest^72^, less inclined to pro-social behavior^73^ and with lower Theory of Mind-related skills^74^. Specularly, this effect was stronger for participants who seek to maintain a positive self-image (i.e., high in social desirability^75^). In keeping with this result, we observed that, when participants fail to adjust their behavior by decreasing their lies toward low-status opponents, they show a weaker tendency to avert their gaze from high-status opponents and maintain it with low-status ones. Deceiving people in need (e.g., those having a low socio-economic status), is usually seen as a violation of moral standards and, for this, less acceptable^11,54^. Thus, both lying less and maintaining less the eye contact with low-status opponents might reflect the burden of moral violation for people who are very much concerned in conveying a positive self-image. This interpretation is supported by previous studies showing how individuals high in social desirability are more sensitive to reputational concerns when deciding to deceive^2^ and how they have a stronger inhibition of the cortical motor preparation when producing self-gain lies^12^.

## Conclusions

All in all, we demonstrate that people’s moral behavior may be influenced by priming values that refer to different political ideologies. More specifically, we report that left-wing ideology activated by ideological words is effective in guiding participants’ behavior during a moral decision-making task. Moreover, the analysis of the participants’ oculomotor behavior reveals how priming affects moral decision making by modifying the allocation of their attention.

## Supplementary information

Supplementary information.

## Data Availability

Data can be found on the repository at this link: https://data.mendeley.com/datasets/kdjvjbd9t7/2.

## AppendixAppendix

**List of right-wing politicians**

Angelino Alfano

Silvio Berlusconi

Renato Brunetta

Maurizio Gasparri

Maria Stella Gelmini

Ignazio La Russa

Matteo Salvini

**List of left-wing politicians**

Pierluigi Bersani

Rosy Bindi

Antonio Di Pietro

Romano Prodi

Matteo Renzi

Walter Veltroni

Nichi Vendola

**List of right-wing words**

Authority

Conservatism

Nationalism

Order

Patriotism

Religiosity

Tradition

**List of left-wing words**

Diversity

Ecology

Hosting

Multiculturalism

Sharing

Secularism

Tolerance

## References

[1] Mazar N, Amir O, Ariely D (2008). The Dishonesty of Honest People: A Theory of Self-Concept Maintenance. J. Mark. Res..

[2] Panasiti, M. S., Pavone, E. F., Merla, A. & Aglioti, S. M. Situational and dispositional determinants of intentional deceiving. *PLoS One* **6** (2011). 10.1371/journal.pone.0019465 PMC3084863 21559381

[3] Panasiti MS, Ponsi G (2017). Commentary: Investigating the Effects of Anger and Guilt on Unethical Behaviour: A Dual-Process Approach. Front. Psychol..

[4] Motro, D. Investigating the Effects of Anger and Guilt on Unethical Decision Making: A Dual-Process Approach, 10.1007/s10551-016-3337-x (2016).

[5] Zhong C-B, Bohns VK, Gino F (2010). Good lamps are the best police: darkness increases dishonesty and self-interested behavior. Psychol. Sci..

[6] Gino F, Mogilner C (2014). Time, Money, and Morality. Psychol. Sci..

[7] Poon K-T, Chen Z, Dewall CN (2013). Feeling entitled to more: ostracism increases dishonest behavior. Pers. Soc. Psychol. Bull..

[8] Schurr, A. & Ritov, I. Winning a competition predicts dishonest behavior. *Proc. Natl. Acad. Sci*. **113** (2016). 10.1073/pnas.1515102113 PMC4763788 26831083

[9] Khan U, Dhar R (2006). Licensing effect in consumer choice. J. Mark. Res..

[10] Brown RP (2011). Moral Credentialing and the Rationalization of Misconduct. Ethics Behav..

[11] Azevedo RT, Panasiti MS, Maglio R, Aglioti SM (2018). Perceived warmth and competence of others shape voluntary deceptive behaviour in a morally relevant setting. Br. J. Psychol..

[12] Panasiti, M. S., Pavone, E. F., Mancini, A., Merla, A. & Aglioti, S. M. The motor cost of telling lies: Electrocortical signatures and personality foundations of spontaneous deception The motor cost of telling lies: Electrocortical signatures and personality foundations of spontaneous deception. 37–41, 10.1080/17470919.2014.934394 (2014). 10.1080/17470919.2014.934394 24979665

[13] Vohs KD, Schooler JW (2008). The value of believing in free will: encouraging a belief in determinism increases cheating. Psychol. Sci..

[14] Shu LL, Gino F, Bazerman MH (2011). Dishonest deed, clear conscience: when cheating leads to moral disengagement and motivated forgetting. Pers. Soc. Psychol. Bull..

[15] Aquino K, Freeman D, Reed A, Lim VKG, Felps W (2009). Testing a social-cognitive model of moral behavior: The interactive influence of situations and moral identity centrality. J. Pers. Soc. Psychol..

[16] Janoff-Bulman R, Sheikh S, Baldacci KG (2008). Mapping moral motives: Approach, avoidance, and political orientation. J. Exp. Soc. Psychol..

[17] Janoff-Bulman R (2009). To Provide or Protect: Motivational Bases of Political Liberalism and Conservatism. Psychol. Inq..

[18] Caprara, G. V., Schwartz, S., Capanna, C., Vecchione, M. & Barbaranelli, C. Personality and Politics: Values, Traits, and Political Choice. *Polit. Psychol*. **27** (2006).

[19] Caprara GV (2017). Basic Values, Ideological Self-Placement, and Voting: A Cross-Cultural Study. Cross-Cultural Res..

[20] Anderson L, Mellor J (2005). Do Liberals Play Nice? The Effects of Party and Political Ideology in Public Goods and Trust Games. Exp. Behav. Econ..

[21] Gjoneska, B., Liuzza, M. T., Porciello, G., Caprara, G. V. & Aglioti, S. M. Bound to the group and blinded by the leader: ideological leader – follower dynamics in a trust economic game. *R. Soc. open Sci*. **6** (2019). 10.1098/rsos.182023 PMC6774964 31598272

[22] Ponsi G, Panasiti MS, Aglioti SM, Liuzza MT (2017). Right-wing authoritarianism and stereotype-driven expectations interact in shaping intergroup trust in one-shot vs multiple-round social interactions. PLoS One.

[23] Celse, J. & Chang, K. Politicians lie, so do I. Psychol. Res. 2 (2017). 10.1007/s00426-017-0954-7 PMC6647169 29189920

[24] Caprara GV, Zimbardo PG (2004). Personalizing politics: A congruency model of political preference. Am. Psychol..

[25] Garzia D (2011). The personalization of politics in Western democracies: Causes and consequences on leader–follower relationships. Leadersh. Q..

[26] Carraro L, Gawronski B, Castelli L (2010). Losing on all fronts: The effects of negative versus positive person-based campaigns on implicit and explicit evaluations of political candidates. Br. J. Soc. Psychol..

[27] Liuzza, M. T., Cazzato, V., Vecchione, M., Crostella, F., M., Caprara, G. V. & Aglioti, S. M. Follow my eyes: the gaze of politicians reflexively captures the gaze of ingroup voters. *PLoS One* **6**, e25117 (2011). 10.1371/journal.pone.0025117 PMC3177843 21957479

[28] Porciello G (2016). Fortunes and misfortunes of political leaders reflected in the eyes of their electors. Exp. Brain Res..

[29] Schepisi, M., Porciello, G., Bufalari, I., Aglioti, S. M. & Panasiti, M. S. Left Threatened by Right: Political Intergroup Bias in the Contemporary Italian Context. *Front. Psychol*. **10** (2019). 10.3389/fpsyg.2019.00026 PMC6353823 30733693

[30] Jost JT, Banaji MR (2004). & Nosek, B. a. A Decade of System Justification Theory: Accumulated Evidence of Conscious and Unconscious Bolstering of the Status Quo John. Polit. Psychol..

[31] Orquin JL, Mueller S (2013). Attention and choice: A review on eye movements in decision making. Acta Psychol. (Amst)..

[32] Fiedler S, Glöckner A, Nicklisch A, Dickert S (2013). Social Value Orientation and information search in social dilemmas: An eye-tracking analysis. Organ. Behav. Hum. Decis. Process..

[33] Fiedler S, Glöckner A (2015). Attention and moral behavior. Curr. Opin. Psychol..

[34] Gino, F., Moore, D. A. & Bazerman, M. H. See No Evil: When We Overlook Other People ‘ s Unethical Behavior And. in Social Decision Making: Social Dilemmas, Social Values, and Ethical Judgments. (eds. Kramer R. M., Tenbrunsel A. E., B. M. P. P. & 2008:241-263.) (2008).

[35] Hochman GUY, Glöckner A, Fiedler S, Ayal S (2016). ‘I can see it in your eyes’: Biased Processing and Increased Arousal in Dishonest Responses. J. Behav. Decis. Mak..

[36] Kastner RM (2010). Moral Judgments and Visual Attention: An Eye- Tracking Investigation. Chrestomathy.

[37] Pittarello A, Motro D, Rubaltelli E, Pluchino P (2016). The relationship between attention allocation and cheating. Psychon. Bull. Rev..

[38] Zettler I, Hilbig BE (2010). Attitudes of the selfless: Explaining political orientation with altruism. Pers. Individ. Dif..

[39] Zettler I, Hilbig BE, Haubrich J (2011). Altruism at the ballots: Predicting political attitudes and behavior. J. Res. Pers..

[40] Emery NJ (2000). The eyes have it: the neuroethology, function and evolution of social gaze. Neurosci. Biobehav. Rev..

[41] Yu H, Duan Y, Zhou X (2017). Guilt in the eyes: Eye movement and physiological evidence for guilt-induced social avoidance. J. Exp. Soc. Psychol..

[42] Festinger L (1962). Cognitive Dissonance. Sci. Am..

[43] Panasiti MS, Cardone D, Pavone EF, Mancini A, Aglioti SM (2016). Thermal signatures of voluntary deception in ecological conditions. Sci. Rep..

[44] Jost JT (2007). Are Needs to Manage Uncertainty and Threat Associated With Political Conservatism or Ideological Extremity? Personal. Soc. Psychol. Bull..

[45] Meston CM, Heiman JR, Trapnell PD, Paulhus DL (1998). Socially desirable responding and sexuality self-reports. J. Sex Res..

[46] Christie R, G. F. Studies In Machiavellianism. (New York: Academi Press, 1970).

[47] Team, R. C. R: A Language and Environment for Statistical Computing. (2018).

[48] Bates, D., Maechler, M., Bolker, B., & Walker, S. lme4: Linear mixed-effects models using Eigen and S4. R package version 1.1–5. (2014).

[49] Garson, G. D. Hierarchical linear modeling. Guide and applications. (Los Angeles: Sage Publications., 2013).

[50] Pinheiro, J. C. & Bates, D. M. Mixed-effects models in S and S-PLUS. (New York: Springer, 2000).

[51] Bates, D., Kliegl, R., Vasishth, S. & Baayen, H. Parsimonious Mixed Models. 1–27, arXiv:1506.04967 (2015).

[52] Bates, D. M. & Pinheiro, J. C. Computational methods for multilevel modelling. *J. Comput. Graph. Stat*. 1–29 (1998).

[53] Liebe U, Tutic A (2010). Status groups and altruistic behaviour in dictator games. Ration. Soc..

[54] Smeets P, Bauer R, Gneezy U (2015). Giving behavior of millionaires. Proc. Natl. Acad. Sci..

[55] Buckholtz, J. W. Social norms, self-control, and the value of antisocial behavior. *Curr. Opin. Behav. Sci.* 122–129 (2015).

[56] Civai C, Ma I (2017). The Enhancement of Social Norm Compliance: Prospects and Caveats. J. Cogn. Enhanc..

[57] Keltner D, Gruenfeld DH, Anderson C (2003). Power, approach, and inhibition. Psychol. Rev..

[58] Dalmaso M, Pavan G, Castelli L, Galfano G (2012). Social status gates social attention in humans. Biol. Lett..

[59] Shepherd, S. V., Deaner, R. O. & Platt, M. L. Social status gates social attention in monkeys. *Curr. Biol*. **16** (2006). 10.1016/j.cub.2006.02.013 16488858

[60] Boukarras, S., Era, V., Aglioti, S. M. & Candidi, M. Modulation of preference for abstract stimuli following competence-based social status primes. Exp. Brain Res. 1–29. 10.1007/s00221-019-05702-z 31832705

[61] Guinote A (2007). Power affects basic cognition: Increased attentional inhibition and flexibility. J. Exp. Soc. Psychol..

[62] Overbeck JR, Park B (2001). When power does not corrupt: Superior individuation processes among powerful perceivers. J. Pers. Soc. Psychol..

[63] Capozzi F (2019). Tracking the Leader: Gaze Behavior in Group Interactions. ISCIENCE.

[64] Capozzi F, Becchio C, Willemse C, Bayliss AP (2016). Followers Are Not Followed: Observed Group Interactions Modulate Subsequent Social Attention. J. Exp. Psychol. Gen..

[65] Dalmaso M, Galfano G, Coricelli C, Castelli L (2014). Temporal Dynamics Underlying the Modulation of Social Status on Social Attention. PLoS One.

[66] Pivetti M, Camodeca M, Rapino M (2016). Shame, Guilt, and Anger: Their Cognitive, Physiological, and Behavioral Correlates. Curr. Psychol..

[67] Terburg D, Aarts H, Van Honk J (2012). Memory and Attention for Social Threat: Anxious Hypercoding-Avoidance and Submissive Gaze Aversion. Emotion.

[68] Gobel MS, Kim HS, Richardson DC (2015). The dual function of social gaze. Cognition.

[69] Jonason PK, Lyons M, Baughman HM, Vernon PA (2014). What a tangled web we weave: The Dark Triad traits and deception. Pers. Individ. Dif..

[70] Jones DN, Paulhus DL (2011). The role of impulsivity in the Dark Triad of personality. Pers. Individ. Dif..

[71] Baughman HM, Jonason PK, Lyons M, Vernon PA (2014). Liar liar pants on fire: Cheater strategies linked to the Dark Triad. Pers. Individ. Dif..

[72] Sakalaki M, Fousiani K (2012). Social Embeddedness and Economic Opportunism: A Game Situation. Psychol. Rep..

[73] Becker JAH, Dan O’Hair H (2007). Machiavellians’ Motives in Organizational Citizenship Behavior. J. Appl. Commun. Res..

[74] Bagozzi RP (2013). Theory of Mind and Empathic Explanations of Machiavellianism: A Neuroscience Perspective. J. Manage..

[75] Paulhus DL (1984). Two-component models of socially desirable responding. J. Pers. Soc. Psychol..

[76] Wickham, H. ggplot2: Elegant Graphics for Data Analysis. (Springer-Verlag New York, 2016).

[77] 2013, I. C. R. IBM SPSS Statistics for Windows, Version 22.0. (Armonk, NY: IBM Corp, 2013).

