# Supplementary material for: Oculomotor behavior tracks the effect of ideological priming on deception
Source: Sci Rep. 2020 Jun 12;10:9555. doi: 10.1038/s41598-020-66151-1 (PMC7293254; doi:10.1038/s41598-020-66151-1)
Supplement: Supplementary file 1 — Supplementary information. [file 41598_2020_66151_MOESM1_ESM.docx]

**Oculomotor behavior tracks the effect of ideological priming on deception**

Michael Schepisi^a,b^, Giuseppina Porciello ^a,b^, Salvatore Maria Aglioti^b,c^ , Maria Serena Panasiti^a,b^

a. Department of Psychology, “Sapienza” University of Rome, Italy.

b. IRCCS, Santa Lucia Foundation, Rome, Italy.

c. Sapienza University of Rome and CNLS @Sapienza Istituto Italiano di Tecnologia, Genova

**Supplementary Materials**

***Cover story***
To avoid the possibility that the participants could find out our experimental hypotheses we set up a cover story that was presented during the instructions phase.
Here we report the part of the instructions relative to the cover story:
*“The aim of this project is to investigate how being involved in a social interaction could influence the perception of different types of stimuli (words, faces etc.). Past research indicates that being involved in specific cognitive or motor processes could affect how we perceive surrounding stimuli. Let’s consider the distracting effect that talking on the phone has on our activities, such as when we have to buy some products at the supermarket.
With this research we aim at expanding these studies by investigating whether interacting with other people -which is a daily process characterizing our lives- could affect how we perceive different types of stimuli.
We will ask you to perform two tasks: one involving the stimuli that you will be observing, and one involving an interaction with other people. The interaction will consist of a simple card game. The stimuli that you will see are words and images selected because of their relevance in advertising. Indeed, advertising has made massive use of testimonials and attractive words in the domains of marketing and mass persuasion.
…At the end of the game you will be asked some questions regarding the images of famous people and words that you have seen at the beginning of each play. Therefore, it is very important for you to pay a lot of attention to those stimuli.”*

*Cover story related tasks*At the end of the experimental procedure participants performed two further tasks.
The first was a recognition task in which they looked at various stimuli and tried to recognize which ones had been shown during the TLCG.
In the second task, only the actual prime stimuli were shown to the participants. For politician stimuli, participants were asked to indicate i) whether they recognized the person by writing his/her surname, ii) which ideology better represented the stimulus by selecting one of four options (*Left-wing, Centre-Left, Centre-Right, Right-wing*) and iii) the emotional valence evoked in them by the stimulus on a 9-point scale (*1=Extremely Negative; 9=Extremely Positive*). For ideological words stimuli, participants were only asked the last two questions.

***Catch Trials***

To make the cover story more believable we inserted some catch trials representing famous people

not belonging to any political category and words not ideologically related.

***List of famous people used as catch trials***

Massimo Boldi (comedian)
Maria De Filippi (Tv Presenter)
Lorenzo Cherubini (Singer)
Alba Parietti (Tv Presenter)

***List of non-ideological words used as catch trials:***Education
Engineering
Personality
Biology

***Payment for participation***

We paid our participants by means of the same procedure employed in all the other studies where the TLCG was used (Panasiti et al., 2011; Panasiti et al., 2014; Panasiti et al., 2016; Azevedo et al., 2018). Specifically, we compensated them with a show-up fee of 15 euro and an additional variable amount ranging between a minimum of 2.50 and a maximum of 10 euro that was based on their performance during the task. Therefore, the more trials they won, the more money they could get. In fact, by losing every trial the participant could win 15 euro; by winning every trial the participant could win 25 euro. When calculating the payment for each participant, we divided the 256 trials in four bands each progressively rewarded with 2.50 euro. For instance, if a participant won from 0 to 64 trials, he/she earned 17.50 euro (15 fixed plus 2.50 euro); if he/she won from 64 to 128 trials, he/she earned 20 euro, and so on so forth. Notably, our participants did not know neither what was the maximum amount of money they could win nor what was the fixed amount of money assigned to single trials. As reported in the rules of the game, we told participants that the payment relative to their opponents would be assigned according to their decisions during the task.

***Stimuli validation procedure and analyses***

Here we report the analyses for the validation and selection of the stimuli (i.e., images of politicians and ideological words) used in the TLCG.

***Politicians’ selection***

Respondents (N=102) were presented with an image (taken from the Internet) of fifty-eight Italian politicians (29 left-wing, 29 right-wing; 23 females, 35 males) and asked to: i) indicate whether they recognized the person by writing his/her surname, and ii) indicate his/her political orientation (*Left-wing, Centre-Left, Centre-Right, Right-wing*). To select the seven left-wing (1 female, 6 males) and seven right-wing (1 female, 6 males) politicians used as stimuli during the TLCG we created an index, ranging from 0 to 1, that resulted as the mean between the percentages of respondents that recognized and assigned to the correct political category each politician.
In bold the politicians that we selected. Some politicians that resulted in having very high scores on the index were not selected because we struggle in finding pictures that we could have easily edit for the purposes of our study.

| \| *Politician* \| *Index of recognition and correct political orientation* \| \| --- \| --- \| \| **BERLUSCONI** \| **0.99** \| \| MUSSOLINI \| 0.96 \| \| **VENDOLA** \| **0.95** \| \| **ALFANO** \| **0.93** \| \| **SALVINI** \| **0.93** \| \| SANTANCHE' \| 0.93 \| \| **RENZI** \| **0.93** \| \| **PRODI** \| **0.93** \| \| **LA RUSSA** \| **0.92** \| \| BOSSI \| 0.92 \| \| **BRUNETTA** \| **0.91** \| \| MELONI \| 0.88 \| \| **GELMINI** \| **0.87** \| \| **BINDI_PO** \| **0.85** \| \| **GASPARRI** \| **0.84** \| \| MARONI \| 0.84 \| \| **BERSANI** \| **0.84** \| \| **DI PIETRO** \| **0.79** \| \| **VELTRONI** \| **0.79** \| \| CARFAGNA \| 0.77 \| \| MARINO \| 0.76 \| \| CASINI \| 0.74 \| \| BONINO \| 0.73 \| \| BOLDRINI \| 0.72 \| \| LETTA \| 0.72 \| \| BOSCHI \| 0.68 \| \| CALDEROLI \| 0.67 \| \| FRANCHESCHINI \| 0.64 \| \| RAZZI \| 0.61 \| \| FORMIGONI \| 0.58 \| \| LUPI \| 0.55 \| \| CIVATI \| 0.51 \| \| CONCIA \| 0.46 \| \| FINOCCHIARO \| 0.44 \| \| MASTELLA \| 0.43 \| \| BORGHEZIO \| 0.41 \| \| FASSINA \| 0.39 \| \| LORENZIN \| 0.32 \| \| MADIA \| 0.31 \| \| POLETTI \| 0.31 \| \| DEL RIO \| 0.30 \| \| DE GIROLAMO \| 0.28 \| \| MOGHERINI \| 0.28 \| \| FITTO \| 0.27 \| \| RAVETTO \| 0.25 \| \| MORETTI \| 0.24 \| \| PRESTIGIACOMO \| 0.22 \| \| GENTILONI \| 0.22 \| \| COMI \| 0.20 \| \| ROMANI \| 0.20 \| \| BIANCOFIORE \| 0.19 \| \| GIANNINI \| 0.17 \| \| VERDINI \| 0.16 \| \| BERNINI \| 0.15 \| \| BONAFE' \| 0.11 \| \| PICERNO \| 0.10 \| |
| --- | --- | --- | --- | --- | --- | --- | --- | --- | --- | --- | --- | --- | --- | --- | --- | --- | --- | --- | --- | --- | --- | --- | --- | --- | --- | --- | --- | --- | --- | --- | --- | --- | --- | --- | --- | --- | --- | --- | --- | --- | --- | --- | --- | --- | --- | --- | --- | --- | --- | --- | --- | --- | --- | --- | --- | --- | --- | --- | --- | --- | --- | --- | --- | --- | --- | --- | --- | --- | --- | --- | --- | --- | --- | --- | --- | --- | --- | --- | --- | --- | --- | --- | --- | --- | --- | --- | --- | --- | --- | --- | --- | --- | --- | --- | --- | --- | --- | --- | --- | --- | --- | --- | --- | --- | --- | --- | --- | --- | --- | --- | --- | --- | --- | --- |

***Images of politicians’ validation and selection***

At least two images for each selected politician were taken from the internet. Each image featured only the face, from a frontal position and with a neutral expression. The neutrality of facial expressions was checked through Microsoft™ API Cognitive Services, an application that assigns a percentage to each of the eight basic emotions (i.e., anger, contempt, disgust, fear, happiness, neutral, sadness, surprise) indicating to what extent each of them is present in the image. The two images with the highest neutrality percentage (> 84%) for each politician were edited with Adobe Photoshop© to delete the background and any contextual information. Images were then included in an online survey and validated by an independent sample of non-Italian people (N= 81, 48 females, age: M= 24.67; SD= ±8.00). Coherently with the API procedure, respondents were asked to indicate, using percentages, to what extent the same eight emotions were expressed by the person in the image. For each stimulus we ran a repeated-measure ANOVA with Emotion as the within-subject factor (eight levels: anger, contempt, disgust, fear, happiness, neutral, sadness, surprise). Post-hoc comparisons were Bonferroni corrected.
Finally, we selected the image in which neutrality was significantly more present than the other emotions. If both images were significantly neutral, we used a two-tailed t-test to choose the more neutral one.
Here we report the Analysis related only to the picture that we used during the task.

*Angelino Alfano*

Results indicated a significant main effect of emotion (F(2.38, 186.33)= 119.96, p<.001). Post-hoc comparisons revealed that neutral emotion was significantly more present than the other seven emotions (M=63.92, SD=±31.61, all Mean differences >48.10, SEs<5.29, all ps<.001).

*Silvio Berlusconi*

Results indicated a significant difference in how the eight emotions are present in this image (F(2.32, 102.39)= 27.29, p<.001). Post-hoc comparisons revealed that neutral emotion was significantly more present than the other seven emotions (M=47.88, SD=±5.57, all Mean differences >26.00, SEs<8.66, all ps<.002).

*Pierluigi Bersani*

Results indicated a significant difference in how the eight emotions are present in this image (F(3.22, 258.24)= 48.42, p<.001). Post-hoc comparisons revealed that neutral emotion was significantly more present than the other seven emotions (M=71.41, SD=±33.21, all Mean differences >29.13, SEs<5.66, all ps<.001).

*Rosy Bindi*

Results indicated a significant difference in how the eight emotions are present in this image (F(2.35, 186.33)= 185.90, p<.001). Post-hoc comparisons revealed that neutral emotion was significantly more present than the other seven emotions (M=63.92, SD=±29.74, all Mean differences >62.78, all SEs<4.71, all ps<.001).

*Renato Brunetta*

Results indicated a significant difference in how the eight emotions are present in this image (F(2.35, 188.16)= 102.60, p<.001). Post-hoc comparisons revealed that neutral emotion was significantly more present than the other seven emotions (M=62.09, SD=±34.49, all Mean differences >52.71, all SEs< 5.12, all ps<.001).

*Antonio Di Pietro*

Results indicated a significant difference in how the eight emotions are present in this image (F(2.47, 195.11)= 121.16, p<.001). Post-hoc comparisons revealed that neutral emotion was significantly more present than the other seven emotions (M=64.25, SD=31.65, SE= ± 3.53, all Mean differences >49.81, all SEs< 5.27, all ps<.001).

*Maurizio Gasparri*

Results indicated a significant difference in how the eight emotions are present in this image (F(2.61, 206.62)= 62.57, p<.001). Post-hoc comparisons revealed that neutral emotion was significantly more present than the other seven emotions (M=54.18, SD=35.46, SE= ± 3.96, all Mean differences >40.65, all SEs< 5.60, all ps<.001).

*Walter Veltroni*

Results indicated a significant difference in how the eight emotions are present in this image (F(2.89, 224.54)= 30.78, p<.001). Post-hoc comparisons revealed that neutral emotion was significantly more present than the other seven emotions (M=37.69, SD=33.64, SE= ± 3.76, all Mean differences >17.25, all SEs< 5.85, all ps<.004).

*Maria Stella Gelmini*

Results indicated a significant difference in how the eight emotions are present in this image (F(2.52, 202.20)= 44.16, p<.001). Post-hoc comparisons revealed that neutral emotion was significantly more present than the other seven emotions (M=41.17, SD=33.27, SE= ± 3.69, all Mean differences >22.59, all SEs< 5.46, all ps<.001).

*Ignazio La Russa*

Results indicated a significant difference in how the eight emotions are present in this image (F(2.18, 174.70)= 92.91, p<.001). Post-hoc comparisons revealed that neutral emotion was significantly more present than the other seven emotions (M=60.74, SD=35.39, SE= ± 3.93, all Mean differences >48.39, all SEs< 5.56, all ps<.001).

*Romano Prodi*

Results indicated a significant difference in how the eight emotions are present in this image (F(2.50, 190.19)= 48.49, p<.001). Post-hoc comparisons revealed that neutral emotion was significantly more present than the other seven emotions (M=49.42, SD=35.32, SE= ± 4.02, all Mean differences >25.90, all SEs< 6.58, all ps<.005).

*Matteo Renzi*

Results indicated a significant difference in how the eight emotions are present in this image (F(1.92, 212.37)= 69.86, p<.001). Post-hoc comparisons revealed that neutral emotion was significantly more present than the other seven emotions (M=75.58, SD=30.56, SE= ± 3.48, all Mean differences >64.28, all SEs< 5.35, all ps<.001).

*Matteo Salvini*

Results indicated a significant difference in how the eight emotions are present in this image (F(2.65, 195.11)= 121.16, p<.001). Post-hoc comparisons revealed that neutral emotion was significantly more present than the other seven emotions (M=55.56, SD=34.04, SE= ± 3.78, all Mean differences >34.81, all SEs< 6.04, all ps<.001).

*Nichi Vendola*

Results indicated a significant difference in how the eight emotions are present in this image (F(2.48, 196.56)= 61.82, p<.001). Post-hoc comparisons revealed that neutral emotion was significantly more present than the other seven emotions (M=51.81, SD=34.44, SE= ± 3.85, all Mean differences >38.31, all SEs< 5.45, all ps<.001).

***Ideological words’ selection***

The ideological words were selected using an online survey that contained a pool of forty-six nouns (23 left-wing, 23 right-wing) taken from political questionnaires (e.g. RWA by Altemeyer and SDO by Sidanius & Pratto). Respondents (N= 82, 47 females, age: M= 29.36; SD= ±9.50) were asked to indicate which ideology each word represented by selecting one of four options: *Left-wing, Right-wing, Both of them, Neither of them*. We selected seven left-wing and seven right-wing words that were consistently assigned to the correct political category.
In bold the words we selected.

| *Word-Italian Word-English Index of correct political orientation*   \| **nazionalismo** \| **nationalism** \| **85%** \| \| --- \| --- \| --- \| \| **conservatorismo** \| **conservatism** \| **84%** \| \| **patriottismo** \| **patriotism** \| **78%** \| \| **ordine** \| **order** \| **74%** \| \| **accoglienza** \| **hosting** \| **71%** \| \| **multiculturalismo** \| **multiculturalism** \| **70%** \| \| **tradizione** \| **tradition** \| **68%** \| \| **autorità** \| **authority** \| **67%** \| \| **condivisione** \| **sharing** \| **67%** \| \| **ecologia** \| **ecology** \| **67%** \| \| **laicità** \| **secularism** \| **64%** \| \| **tolleranza** \| **tolerance** \| **64%** \| \| **diversità** \| **diversity** \| **63%** \| \| **religiosità** \| **religiosity** \| **63%** \| \| obbedienza \| obedience \| 60% \| \| uguaglianza \| equality \| 60% \| \| collettività \| collectivity \| 59% \| \| controllo \| control \| 59% \| \| ridistribuzione \| redistribution \| 59% \| \| solidarietà \| solidarity \| 57% \| \| cooperazione \| cooperation \| 56% \| \| dominanza \| dominance \| 54% \| \| gerarchia \| hierarchy \| 52% \| \| preservazione \| preservation \| 52% \| \| sicurezza \| safety \| 52% \| \| individualità \| individualism \| 51% \| \| cambiamento \| change \| 48% \| \| conformismo \| comformism \| 46% \| \| assistenza \| assistance \| 43% \| \| pace \| peace \| 43% \| \| protezione \| protection \| 42% \| \| progresso \| progress \| 39% \| \| dogmatismo \| dogmatism \| 37% \| \| competizione \| competition \| 36% \| \| libertà \| freedom \| 36% \| \| riforma \| reform \| 35% \| \| flessibilità \| flexibility \| 34% \| \| stabilità \| stability \| 34% \| \| innovazione \| innovation \| 31% \| \| aiuto \| help \| 30% \| \| castità \| chastity \| 30% \| \| certezza \| certainty \| 30% \| \| convenzione \| convention \| 29% \| \| liberalismo \| liberalism \| 26% \| \| mobilità \| mobility \| 26% \| \| meritocrazia \| meritocracy \| 24% \| |
| --- | --- | --- | --- | --- | --- | --- | --- | --- | --- | --- | --- | --- | --- | --- | --- | --- | --- | --- | --- | --- | --- | --- | --- | --- | --- | --- | --- | --- | --- | --- | --- | --- | --- | --- | --- | --- | --- | --- | --- | --- | --- | --- | --- | --- | --- | --- | --- | --- | --- | --- | --- | --- | --- | --- | --- | --- | --- | --- | --- | --- | --- | --- | --- | --- | --- | --- | --- | --- | --- | --- | --- | --- | --- | --- | --- | --- | --- | --- | --- | --- | --- | --- | --- | --- | --- | --- | --- | --- | --- | --- | --- | --- | --- | --- | --- | --- | --- | --- | --- | --- | --- | --- | --- | --- | --- | --- | --- | --- | --- | --- | --- | --- | --- | --- | --- | --- | --- | --- | --- | --- | --- | --- | --- | --- | --- | --- | --- | --- | --- | --- | --- | --- | --- | --- | --- | --- | --- | --- |

Furthermore, we checked that there were no differences in characters and syllabic length and in lexical frequency between left and right-wing words (CoLFIS, Bertinetto et al., 2005. Corpus e Lessico di Frequenza dell'Italiano Scritto (CoLFIS); <http://linguistica.sns.it/CoLFIS/Home.htm>).

We ran three independent t-tests on each of these variables and found no difference between left and right-wing words.
Characters length: left-wing (M_left_=10.57, SD_left_=3.30); right-wing words (M_right_=10.57, SD_right_=±2.93) (t(12)=0.00, p=1).
Syllabic length: left-wing (M_left_=4.57, SD_left_=1.27); right-wing words (M_right_=4.42, SD_right_=±.97) (t(12)=-.23, p=.81).
Lexical frequency: left-wing (M_left_=28.14, SD_left_=27.56); right-wing words (M_right_=167.14, SD_right_=±224.83) (t(12)=1.62, p=.15).

***Images of the participants’ opponents in the TLCG***

We selected sixteen free-licensed images (8 females, 8 males) from the internet with the purpose of representing the fictitious opponents in the card game. The images depicted only the person’s face in a frontal position and with the gaze directed towards the participants. Images were then edited to delete any contextual information and assigned to two groups, one of high status and one of low status (4 females, 4 males for each group), that were counterbalanced across subjects. All the Stimuli (i.e., politicians, ideological words and opponents) were resized and implemented in the adapted version of the “Temptation to Lie Card Game”.

***Counterbalancing procedure within the TLCG***

Blocks with the same type of stimuli (e.g., words or politicians) were presented in pairs, namely we presented one type of stimuli (e.g., pictures of politicians) in the first two blocks and the other type (e.g., words) in the other two.
The following elements were counterbalanced across subjects: the meaning of the symbols on the cards (i.e., *winning/losing card or high/low status opponent*), the buttons on the keyboard used for indicating the decision (*V* and *M* for *truth/lie)* and the pictures of the fictitious opponents (i.e., high status opponents for some participants were low status opponents for other participants).
The card presentation order was counterbalanced within subjects: at the beginning of each block, participants were informed that half the trials would feature the outcome card on top and the status card on bottom, while in the other half of trials this order would be reversed

***AOI definition***
Here we report a figure and the description of how we defined the AOIs.


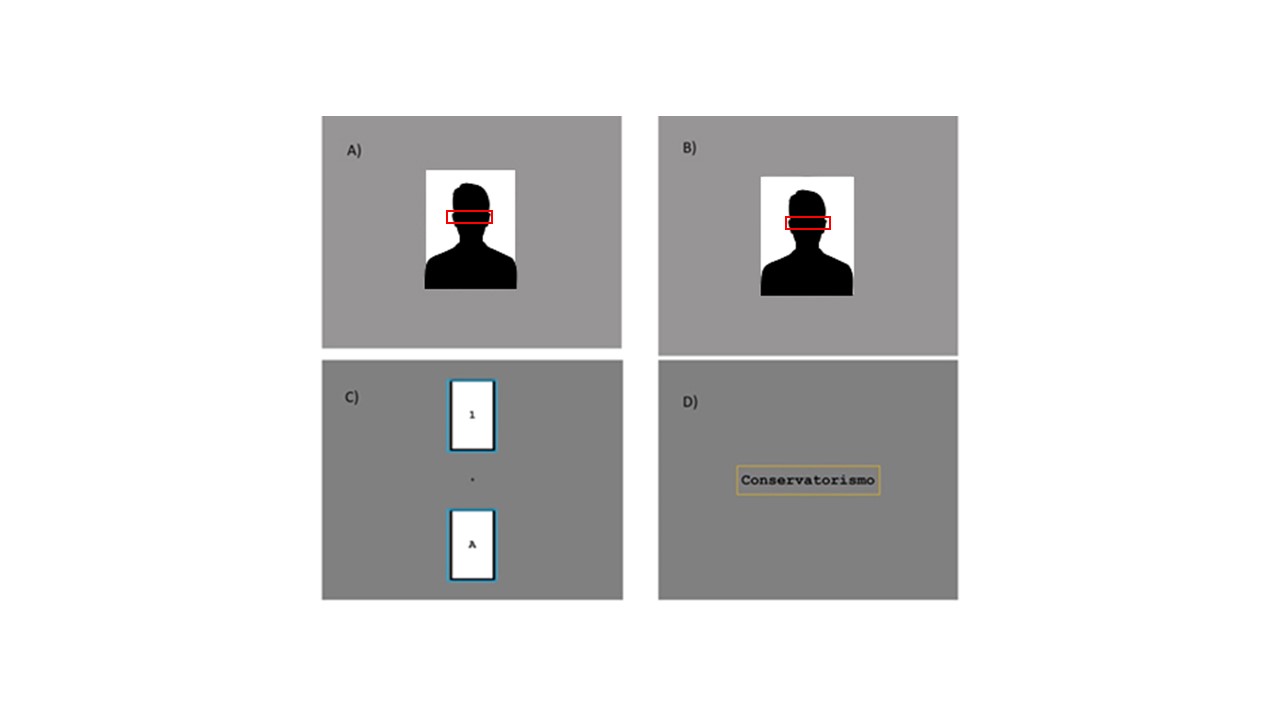


We created AOIs in the software IViewX by superimposing a rectangle on the area that included the elements we were interested in (such as eyes, words, cards etc.). The result was a box having certain X-Y coordinates that corresponded to the area in which the element of interest was located.
In the case of Politicians (panel A) and Opponents’ (panel B) eyes the AOI the rectangle included the eyes of the person represented in the image. In the present figure the images of the politician and the opponent are presented anonymously.
In the case of the cards representing the outcome and the status of the opponents (panel C) the rectangle included the portion of the image in which the two cards were located.
In the case of the words (panel D) the rectangle included a portion of space that could contain all the words in their different lengths.
For the analysis we then extracted participants’ fixations duration that fell in the X-Y coordinates that were delimiting each AOI.

***Mixed-models statistical analyses***

1. Lie-Truth ~ (Political orientation of the priming + Opponent’s Status + Priming type + Outcome of the game + Political orientation of the priming : Opponent’s Status + Political orientation of the priming : Priming type + Opponent’s Status : Priming type + Political orientation of the priming : Outcome of the game + Opponent’s Status : Outcome of the game |Subject) + Political orientation of the priming * Opponent’s Status * Priming type * Outcome of the game
2. Fixation Outcome AOI/Status AOI ~ (Priming type * Political orientation of the priming |Subject) + Priming fixations duration * Priming type * Political orientation of the priming
3. Lie-Truth ~ (Opponent’s Status + Outcome of the game |Subject) + Opponent’s Status * Outcome of the game * Personal first
4. Fixations toward opponents eyes ~ Lie/Truth + Outcome of the game + Opponent’s Status + Political orientation of the priming + Priming type + Lie/Truth : Outcome of the game + Lie/Truth : Opponent’s Status + Outcome of the game : Opponent’s Status + Lie/Truth : Political orientation of the priming + Lie/Truth : Priming type + Outcome of the game : Priming type + Opponent’s Status : Priming type + Political orientation of the game : Priming type + Lie/Truth : Outcome of the game : Opponent’s Status + Lie/Truth : Outcome of the game : Political orientation of the priming + Lie/Truth : Opponent’s Status : Political orientation of the priming + Outcome of the game : Opponent’s Status : Political orientation of the priming + Lie/Truth : Outcome of the game : Priming type + Lie/Truth : Opponent’s Status : Priming type |Subject) + Lie/Truth * Outcome of the game * Opponent’s Status * Political orientation of the priming * Priming type

***Participants’ socio-economic status***

The socio-economic status of our participants was measured at the end of the experiment by asking them the question “compared to the employees you played with, where would you locate your socio-economic status?”. Participants could give their answer by means of a VAS ranging from 0 to 100, where 0 indicated an extremely low and 100 an extremely high socio-economic status (M=50.23, SD=14.12).
Then, as for personality traits, we correlated this measure in the analysis with the BLUPs (i.e., best linear unbiased predictors), deriving from the estimates of the significant interactions of our models of interest. We found no significant correlations with any of the BLUPs (Rs <.27, ps > .09), indicating that participants’ socio-economic status was not associated with any of our significant effects.

***Correlation matrix***

The matrix reports the correlation between the dependent variables of each model (number of lies vs. truth, Fixations duration towards Outcome AOI/Status AOI, Fixations duration toward opponents eyes) and the covariates of interest (Fixations duration toward the priming stimuli, the Machiavellianism scale and the Balanced Inventory of Desirable Responding scale).
All Pearson’s correlations are two-tailed. *p<.05, **p<.01

| Variable | 1 | 2 | 3 | 4 | 5 | 6 |
| --- | --- | --- | --- | --- | --- | --- |
|  |  |  |  |  |  |  |
| 1. Lie_Truth |  |  |  |  |  |  |
|  |  |  |  |  |  |  |
| 2. Fixations_Priming | -.12 |  |  |  |  |  |
|  |  |  |  |  |  |  |
| 3.Fixations_Cards | .09 | .08 |  |  |  |  |
|  |  |  |  |  |  |  |
| 4. Fixations_Opp_Eyes | -.03 | .31 | .03 |  |  |  |
|  |  |  |  |  |  |  |
| 5. MACH IV | -.06 | .18 | .17 | .22 |  |  |
|  |  |  |  |  |  |  |
| 6. BIDR | -.09 | -.41* | -.13 | -.31 | -.60** |  |
|  |  |  |  |  |  |  |
|  |  |  |  |  |  |  |
|  |  |  |  |  |  |  |
|  |  |  |  |  |  |  |
